# Meaning in Life Mediates Associations Between Gratitude, Forgiveness, Spirituality, and Mental Health in Postgraduate Students

**DOI:** 10.3390/ejihpe16020025

**Published:** 2026-02-13

**Authors:** Muhammad Adeeb, Mariny Abdul Ghani, Azlin Hilma Hillaluddin, Luca Flesia

**Affiliations:** 1 Department of Professional Psychology, Bahria University Lahore Campus, Lahore 54600, Pakistan; 2 School of Applied Psychology, Social Work and Policy, University of Utara, Sintok 06010, Malaysia; 3 Azienda Ulss 6 Euganea, Veneto Region, National Health Service, 35131 Padova, Italy

**Keywords:** mental health, gratitude, forgiveness, spirituality, meaning in life

## Abstract

Mental health concerns are increasingly prevalent among postgraduate students, who face academic, social, and career pressures. Although research on student mental health is expanding, less is known about the psychological resources that support well-being in postgraduate learners. Meaning in life has been identified as a key psychological resource that helps individuals interpret challenges and maintain coherence and well-being, and recent research highlights the contribution of self-transcendent traits such as gratitude, forgiveness, and spirituality in fostering meaning in life. However, empirical evidence on these interrelationships remains limited. This study involves 1527 Pakistani postgraduate students (M = 795; mean age = 24.89 years) recruited through multistage random sampling from ten public universities in Punjab. Participants completed the Gratitude Questionnaire, Heartland Forgiveness Scale, Spirituality Scale, Meaning in Life Questionnaire, and Mental Health Inventory (assessing psychological well-being and psychological distress). Correlation analyses showed that gratitude, forgiveness, and spirituality were positively associated with psychological well-being and negatively associated with psychological distress. Structural equation modeling (SEM) further indicated that these traits predicted mental health both directly and indirectly, with meaning in life serving as significant partial mediator. Overall, the findings highlight the central role of meaning in life in linking self-transcendent traits to mental health among postgraduate students and suggest important implications for culturally sensitive, university-based mental health initiatives.

## 1. Introduction

Mental health is emerging as a critical issue in the public health and educational-preventive field: the prevalence of mental health issues is rising, becoming one of the most pressing global health concerns facing modern society (Hussain et al., 2018). Within this field, university students represent a particularly important sub-population (Vargas-Huicochea et al., 2022; Wahed & Hassan, 2017). Recent evidence shows that university students report poorer mental health than their non-university peers, with increasing severity over time due to pandemic-related and socio-cultural stressors (Blanco et al., 2008; Chen & Lucock, 2022; Levecque et al., 2017).

In this sense, postgraduate students are at even greater risk, as they experience a specific “in-between condition”, situated between academic life, the approaching completion of their studies, and the transition into the job market. This transitional phase heightens uncertainty and future-related pressure, often becoming a source of anxiety (Morris, 2021).

Mental health is broadly defined as the capacity for positive and effective mental functioning (Hersi et al., 2017), a state of well-being enabling individuals to cope with life stressors (Lal et al., 2014). According to Veit and Ware (1983), it includes both the dimensions of psychological well-being and psychological distress.

To understand the psychological resources that buffer these challenges, recent research has focused on existential constructs such as meaning in life, defined as people’s sense of coherence, purpose, and significance (Heintzelman & King, 2014; Steger, 2022), or the capacity to make sense of one’s life and perceive predictability and consistency (Steger, 2022). Meaning in life is recognized as a key factor in mental functioning, predicting both well-being and reduced distress (Steger, 2022). Among university students, it serves as a protective factor against psychosomatic symptoms (Wang et al., 2016) and depression (Carreno et al., 2020; Huo et al., 2020). Within existential and positive psychological frameworks, meaning in life is considered a central mechanism enabling individuals to navigate adversity and sustain well-being (Steger, 2022). According to this perspective, self-transcendent qualities—such as the ability to focus beyond one’s immediate needs, appreciate what one has, and find purpose in life—enhance resilience and protect against psychological distress.

Gratitude, forgiveness, and spirituality can be conceptualized as self-transcendent traits that orient individuals beyond the self and foster connection, acceptance and sense of purpose. These traits are hypothesized to enhance meaning in life, thereby supporting psychological well-being and mitigating distress.

Gratitude has been described as a self-transcendent emotion and attitude, a life orientation toward perceiving and appreciating good things in one’s life and positive aspects of the world, even in difficult circumstances (McCullough et al., 2008; Stellar et al., 2017). Gratitude has been linked to enhanced well-being and reduced symptoms of depression, anxiety, and stress (Bohlmeijer et al., 2021; Cregg & Cheavens, 2021). Research among students consistently shows these associations (Guse et al., 2019; Măirean et al., 2019; Mason, 2019; Sapmaz et al., 2016). 

Forgiveness, as defined by Rye and Pargament (2002), involves the process of letting go of negative feelings (e.g., resentment), thoughts (e.g., retribution), and behaviors (e.g., hostility), that can be directed toward oneself (self-forgiveness), others, or situations (Thompson et al., 2005). It has consistently been found to be associated with reduced distress and greater well-being (Fincham & May, 2021), also among university students Kravchuk (2022).

Spirituality is considered a universal aspect of human experience through which individuals seek ultimate meaning, purpose, and transcendence, experiencing connectedness to themselves, others, nature, and the significant or sacred (Long et al., 2024; Wüthrich-Grossenbacher, 2024). It is linked to psychological resilience, well-being (Garssen et al., 2021; Jeny & Varghese, 2013) and mental health (Holder et al., 2016; Litalien et al., 2022), also among university students (de Diego Cordero et al., 2019; M. Khan, 2015; Negi et al., 2021). While closely related, spirituality and meaning in life are conceptually distinct constructs, with spirituality reflecting a broader existential orientation grounded in transcendence and connectedness, and meaning in life capturing the subjective appraisal of purpose, coherence, and significance (Bernard et al., 2017). 

Previous studies have found that meaning in life is associated with gratitude, forgiveness, and spirituality (Oriol et al., 2020; Głaz, 2019; Yoon et al., 2021) and mediates relationships between positive traits and mental health (Halama & Dedova, 2007; Karaman et al., 2020; Zhou & Xu, 2019; Datu & Mateo, 2015; Sargent, 2015).

Despite these findings, little research has examined the relationship between meaning in life and self-transcendent traits among postgraduate students—a population facing unique transitional stressors and cultural pressures. 

This is especially relevant in Pakistan, where mental health among university students remains a neglected field (Saleem et al., 2013), and psychological distress is widespread (Ghayas et al., 2014; Jibeen, 2016; Bibi et al., 2021; M. N. Khan et al., 2021). Socio-cultural changes in postgraduate education in Pakistan—such as relocation, financial strain, and shifts in academic expectations—further intensify these challenges (Kaur & Sidhu, 2009). 

Notably, no studies have investigated the joint contribution of gratitude, forgiveness, and spirituality to mental health through meaning in life in this population. Building upon prior studies that identified meaning in life as a mediator between positive traits (e.g., gratitude, spirituality, resilience) and mental health outcomes (Datu & Mateo, 2015; Karaman et al., 2020), the present study extends existential positive psychology by testing meaning in life as a mediator linking three self-transcendent traits to mental health within a culturally distinctive and understudied postgraduate context. This study offers a unique theoretical contribution by integrating three self-transcendent traits within a single model and testing meaning in life as the shared psychological mechanism linking them to mental health. This integrated approach has not yet been examined in postgraduate populations, particularly within non-Western contexts.

Based on the literature reviewed, the following hypotheses were proposed:

**H1.**  *Higher levels of gratitude, forgiveness, and spirituality will be associated with higher levels of psychological well-being and overall mental health among postgraduate university students.*

**H2.**  *Higher levels of gratitude, forgiveness, and spirituality will be associated with lower levels of psychological distress among postgraduate university students.*

**H3.**  *Meaning in life will mediate the relationship between gratitude, forgiveness, and spirituality and mental health among postgraduate university students.*

## 2. Materials and Methods

### 2.1. Participants

In the current study, a total of 1527 postgraduate university students were recruited using a probability-based multistage random sampling technique. The sampling process was conducted in four stages. In the first stage, stratified random sampling was used to select ten public sector universities from the nine administrative divisions of the Punjab province, Pakistan. These included: Lahore Division: University of the Punjab and Lahore College for Women University; Faisalabad Division: Government College University Faisalabad; Rawalpindi Division: Arid Agriculture University; Gujranwala Division: University of Gujrat; Sahiwal Division: University of Sahiwal; Bahawalpur Division: The Islamia University of Bahawalpur; Multan Division: Bahauddin Zakariya University; Sargodha Division: University of Sargodha; Dera Ghazi Khan Division: Ghazi University. Two universities were selected from the Lahore Division, the capital and most densely populated region of Punjab. According to the Economic Survey of Pakistan, approximately 60% of the country’s population (110 million people) resides in Punjab, while the remaining 40% lives across the other three provinces (Dar & Wasti, 2017). 

As larger strata require proportionally larger samples (Scheaffer et al., 1990), the sample was drawn from ten universities within Punjab. In the second stage, stratified random sampling was applied to select three academic faculties from each university. These were: Faculty of Science; Faculty of Arts and Humanities; and Faculty of Social Sciences. These faculties were selected from a total of six available faculties (including Medical, Agriculture, and Engineering) to enhance generalizability across disciplines most relevant to the study. In the third stage, three academic departments were selected from each of the three chosen faculties within each university, resulting in nine departments per university. The departments selected were: Faculty of Science: Mathematics, Physics, Chemistry; Faculty of Arts and Humanities: English, Urdu, Islamic Studies; and Faculty of Social Sciences: Psychology, Economics, and Social Work/Sociology.

In the fourth and final stage, simple random sampling was employed to select 20 postgraduate students (Master’s and PhD level) from each department. The initial target sample was 1800 participants. However, after discarding 273 questionnaires due to incomplete responses or missing data, the final valid sample consisted of 1527 postgraduate students. This study received ethical approval from the Ethical Committee of University Utara Malaysia (Reference No.: SAPSP/Off-2021/903715).

The rationale for choosing public sector universities lies in the fact that over 80 percent of students pursuing higher education are enrolled in these institutions (Mangi et al., 2011). Typically, public sector universities attract students from all socioeconomic backgrounds, while private universities often cater to students from middle and upper socioeconomic statuses due to the higher fees. In 2018, according to Pakistan’s most recently published Education Statistics (2016–2017), the total postgraduate enrollment of students in universities located in Pakistan were 1.463 million. Of these, 1.192 million students (81%) studied in public universities, whereas 0.270 million (19%) studied in private universities (Pakistan Education Statistics 2016–17, 2018). Therefore, only public sector universities were selected for data collection. 

### 2.2. Measures 

#### 2.2.1. Demographic Sheet

A demographic information sheet was used to collect participants’ background details, including: age (continuous variable); gender (male, female); education level (Master’s, PhD); home residence (rural or urban); family system (a joint family included living with grandparents and uncles/aunts, while a nuclear family included living only with parents); monthly family income (continuous variable); and marital status (single, married or divorced). A detailed table summarizing the demographic profile of the participants is provided in the Supplementary Materials.

#### 2.2.2. Gratitude Questionnaire (GQ)

The Gratitude Questionnaire (GQ-6), developed by McCullough et al. (2001), was used to assess dispositional gratitude. It consists of six items, rated on a 7-point Likert scale ranging from 1 = strongly disagree to 7 = strongly agree (example item: “I have so much in life to be thankful for”). Items 1, 2, 4, and 5 are positively keyed, while items 3 and 6 require reverse scoring. Total scores range from 6 to 42, with higher scores indicating greater gratitude (McCullough et al., 2001). In a past study with Pakistani university students’ sample, the internal consistency was found to be good range (Cronbach’s α = 0.85) (Fatima et al., 2022); thus, it was considered appropriate for use in the present study.

#### 2.2.3. Heartland Forgiveness Scale (HFS)

The Heartland Forgiveness Scale (HFS), developed by Thompson et al. (2005), was used to measure individuals’ tendencies to forgive in this study. The scale comprises 18 items across three subscales: Self-forgiveness (Items 1–6); Forgiveness of others (Items 7–12); Forgiveness of situations (Items 13–18). Each item is rated on a 7-point Likert scale from 1 = almost always false of me to 7 = almost always true of me (example item: “Although I feel badly at first when I mess up, over time I can give myself some slack”). Internal consistency was acceptable for all subscales and the overall scale: Self-forgiveness (α = 0.75); Forgiveness of others (α = 0.78); Forgiveness of situations (α = 0.77); Total HFS (α = 0.86) (Thompson et al., 2005). In a past study with Pakistani university students’ sample, the internal consistency was found to be acceptable range (Cronbach’s α = 0.78) (Hermaen & Bhutto, 2020); thus, it was considered appropriate for use in the present study.

#### 2.2.4. Spirituality Scale (SS)

The Spirituality Scale (SS), developed by Parsian and Dunning (2009), was used to assess spirituality among participants. The instrument consists of 23 items and is divided into three subscales: Self-discovery (Items 1–4); Relationships (Items 5–10); Eco-awareness (Items 11–23). Responses are measured on a 6-point Likert scale ranging from 1 = strongly disagree to 6 = strongly agree (example item: “My spirituality gives me inner strength”). Higher scores indicate a greater level of spirituality. The total score ranges from 23 to 138. Internal consistency was excellent (Cronbach’s α = 0.94) (Parsian & Dunning, 2009). In a past study with Pakistani university students’ sample, the internal consistency was found to be good range (Cronbach’s α = 0.84) (Subhan et al., 2024); thus, it was considered appropriate for use in the present study.

#### 2.2.5. The Meaning in Life Questionnaire (MLQ)

The Meaning in Life Questionnaire (MLQ), developed by Steger et al. (2006), was used to assess participants’ perceptions of meaning in life. The scale includes 10 items, rated on a 7-point Likert scale ranging from 1 = absolutely untrue to 7 = absolutely true (example item: “I understand my life’s meaning”). The scale measures two dimensions: Presence of meaning and Search for meaning. Reverse scoring is required for some items before computing the total score. The score range is 10 to 70, with higher scores indicating a greater perceived meaning in life. Internal consistency reliability ranged from α = 0.81 to 0.92 (Steger et al., 2006). In a past study with Pakistani university students’ sample, the internal consistency was found to be excellent range (Cronbach’s α = 0.90) (Muhammad et al., 2020); thus, it was considered appropriate for use in the present study.

#### 2.2.6. Mental Health Inventory (MHI)

The Mental Health Inventory (MHI), developed by Veit and Ware (1983), was used to assess overall mental health. The scale consists of 38 items, divided into two subscales: Psychological distress (22 items; example item: “How much of the time have you felt lonely during the past month?”); Psychological well-being (16 items; example item: “How much time, during the past month, did you feel relaxed and free from tension?”). Items are rated on a 6-point Likert scale ranging from 1 = all of the time to 6 = none of the time. Scores range from: 22 to 132 for psychological distress; 16 to 96 for psychological well-being. To compute an overall mental health score, psychological distress items must be reverse scored, as higher values indicate worse mental health. Internal consistency was excellent, with Cronbach’s α ranging from 0.92 to 0.96 (Veit & Ware, 1983). In a past study with Pakistani university students’ sample, the internal consistency was found to be excellent range (Cronbach’s α = 0.95 to 0.96) (M. J. Khan et al., 2015); thus, it was considered appropriate for use in the present study.

### 2.3. Research Procedure

Data collection took place between October 2021 and February 2022. Prior to data collection, participants were informed that their responses would remain confidential and would be used solely for research purposes. After obtaining formal ethical approval, the researcher provided a detailed oral briefing to explain the objectives of the study. All participants were approached at the selected public universities across Pakistan. Self-report questionnaires were used for data collection. These were distributed by hand by the researcher to ensure that each participant received the materials and to allow for immediate completion. Hand delivery also allowed the researcher to offer clarification if participants had questions or required additional guidance during the process. Upon receiving the questionnaires, participants were given an informed consent form, and participation was entirely voluntary. Participants were assured of their right to withdraw from the study at any time without any penalty. To ensure ethical compliance, all identifying information was kept strictly confidential. Furthermore, participants who experienced any discomfort or psychological distress were provided with access to free counseling support. To reduce potential bias associated with in-person, self-administered data collection, the researchers provided standardized written instructions, ensured anonymity, and maintained a neutral presence throughout administration. These procedures helped minimize social-desirability bias and unintended researcher effects on participants’ responses. 

It took approximately 25 to 35 min to complete the questionnaires.

### 2.4. Normality Test

Before investigating Covariance Based Structural Equation Modeling (CB-SEM) through AMOS (25V), data normality was assessed through normality and outlier tests in AMOS. It is recommended that data meet the assumption of normality when applying CB-SEM (Hair et al., 2014). Accordingly, two approaches were employed: assessment of skewness and kurtosis for each variable, and examination of multivariate outliers. First, skewness and kurtosis values were calculated. Kline (2023) suggested that a standardized kurtosis value of 3 indicates a normal distribution; values above this threshold indicate positive kurtosis, whereas values below indicate negative kurtosis. According to these criteria, the kurtosis values in the present study fell within an acceptable range (−0.93 to 0.09), with none exceeding the recommended threshold, thereby supporting univariate normality. Similarly, West et al. (1995) suggested that substantial departures from normality are indicated when the absolute value of skewness exceeds 7. In the present study, skewness values for all variables were below the criteria (−0.89 to 0.21), further confirming univariate normality. Second, multivariate normality was examined by assessing the presence of multivariate outliers using, Mardia (1974) coefficient of multivariate kurtosis. The normalized estimate of multivariate kurtosis was 2.02, with acritical ratio of 2.34. Multivariate normality is assessed based on a large-sample theory, in which this estimate is assumed to follow a standard normal distribution (Bentler & Wu, 2005). Large values indicate significant positive kurtosis. Bentler and Wu (2005) recommended that values greater than 5.00 indicate non-normality. In the present study, the obtained values were below this threshold, supporting the assumption of multivariate normality.

### 2.5. Data Analysis

Data analysis was carried out in two main stages. In the first stage, the measurement model was assessed using Confirmatory Factor Analysis (CFA) through AMOS to evaluate the reliability and validity of the study instruments. As many researchers have used AMOS for CFA in their studies (Hasim et al., 2024; Sureshchandar, 2023). CB-SEM was employed because the main objective of the study was confirmation of hypothesized relationships, rather than prediction or exploratory model development (Hair et al., 2019; Kline, 2023). CB-SEM is particularly suitable for confirmatory research designs where them emphasis is on evaluating whether the proposed theoretical model adequately fits the observed data (Bollen, 1989; T. A. Brown, 2015). Meanwhile, additional advantage of CB-SEM is its ability to assess global model fit using well-established goodness of fit indices, such as Chi-Square statistics, CFI, TLI, and RMSEA, which are central to theory-driven model evaluation (Hair et al., 2019; Kline, 2023). These indices allow researchers to determine how well the overall covariance structure implied by the theoretical model corresponds to the empirical data (T. A. Brown, 2015). 

Furthermore, the sample size and data normality of the study met the assumptions required for CB-SEM (Dash & Paul, 2021; Hair et al., 2019; West et al., 1995). As Çelik and Yılmaz (2016) recommended sample size should be in range of 200–500, while the sample size of this research meets this assumption. Finally, CB-SEM is well suited for testing mediation model grounded in theory, as it allows for the simultaneous role examined in the study, CB-SEM was considered more appropriate than variance-based alternatives such as partial least square structural equation modelling (PLS-SEM).

To examine relationships among variables, Pearson’s product-moment correlation coefficients were computed using SPSS (27.0). In the second stage, the structural model was tested to evaluate the study hypotheses using CB-SEM with AMOS (Mia et al., 2019).

## 3. Results

### 3.1. Measurement Model 

The measurement model was analyzed for reliability and validity. Using CB-SEM, the complete fit model yielded a χ^2^ value of 14,495.15 (*p* < 0.01). Model fit indices were employed to assess the adequacy of the data fit with the established model. This evaluation involved a single-step process in which both absolute and relative fit indices were computed, including the Goodness of Fit Index (GFI), Comparative Fit Index (CFI), Normed Fit Index (NFI), Root Mean Square Error of Approximation (RMSEA), and Standardized Root Mean Square Residual (SRMR). The absolute model fit was assessed using the chi-square test, taking into account sample size accuracy and the total number of parameters. Previous researchers often use multiple descriptive fit measures to ensure a comprehensive assessment of the model fit. In this context, Azzi et al. (2023) recommended specific criteria: a χ^2^/df ratio between 1 and 3, even values less than 5 indicate a reasonable fit (Hu & Bentler, 1999) RMSEA and SRMR values of 0.08 or smaller, and values exceeding 0.90 for CFI, TLI, and GFI as indicative of a good fit. The initial model demonstrated an RMSEA of 0.042 and an SRMR of 0.038, while the CFI, GFI, and TLI values were 0.868, 0.843, and 0.865, respectively. The χ^2^/df ratio was 3.718. Based on these descriptive fit measures, the model exhibited a less than strong fit.

After modifying the initial model, the fit indices (see Table 1) demonstrated significant improvement, with an RMSEA of 0.031 and an SRMR of 0.029. The values for CFI, GFI, and TLI were 0.912, 0.901, and 0.924, respectively, and the χ^2^/df ratio stood at 2.934. These descriptive fit measures indicate that the model exhibited a very strong fit, showing a robust alignment with the data. Following the satisfactory fit of the model, the factor loadings were confirmed, which are expected to exceed 0.50 for acceptability (Rita et al., 2019) and all exceeded 0.60 in this study and majority were above 0.70. The construct reliability was evaluated using Cronbach’s alpha (α) and composite reliability (CR). The values obtained were as follows: Gratitude (α = 0.873, CR = 0.870), Forgiveness (α = 0.935, CR = 0.839), Self-Forgiveness (α = 0.879, CR = 0.876), Others-Forgiveness (α = 0.800, CR = 0.881), Situational-Forgiveness (α = 0.878, CR = 0.876), Spirituality (α = 0.941, CR = 0.892), Self-Discovery (α = 0.922, CR = 0.823), Relationships (α = 0.865, CR = 0.866), Eco-Awareness (α = 0.930, CR = 0.884), Meaning in Life (α = 0.918, CR = 0.914), Mental Health (α = 0.922, CR = 0.858), Psychological Wellbeing (α = 0.918, CR = 0.885), and Psychological Distress (α = 0.932, CR = 0.877). According to Hair et al. (2014), Cronbach’s alpha values should exceed 0.70, while CR values should surpass 0.80. Consequently, both Cronbach’s alpha and CR values were deemed acceptable, as they surpassed these threshold values (Sarstedt & Cheah, 2019). 

Furthermore, the assessment of construct validity utilized the average variance extracted (AVE). The AVE values were as follows: Gratitude (AVE = 0.531), Forgiveness (AVE = 0.545), Self-Forgiveness (AVE = 0.541), Others-Forgiveness (AVE = 0.552), Situational-Forgiveness (AVE = 0.543), Spirituality (AVE = 0.541), Self-Discovery (AVE = 0.537), Relationships (AVE = 0.520), Eco-Awareness (AVE = 0.521), Meaning in Life (AVE = 0.519), Mental Health (AVE = 0.503), Psychological Wellbeing (AVE = 0.513), and Psychological Distress (AVE = 0.506). All AVE values met the threshold of 0.50 recommended by (Sarstedt et al., 2011). Additionally, the study checked for multicollinearity concerns using the variance inflation factor (VIF) test. Following the recommendations of Hair et al. (2014), the VIF values should not exceed 5. The VIF values for all constructs were below this threshold, indicating the absence of multicollinearity concerns in the dataset.

#### Discriminant Validity 

Discriminant validity was assessed using the method proposed by Fornell and Larcker (1981a). According to the criteria defined by Fornell and Larcker (1981b), discriminant validity is established when the square root of the AVE for each construct exceeds the correlations between constructs. In the present study, the square roots of the AVEs indeed exceeded the inter-construct correlations, indicating that all measurement constructs were suitable for inclusion in the structural model.

### 3.2. Bivariate Correlation (Hypotheses 1–2)

The results of Table 2 indicate the results of Pearson Product Moment Correlation. Gratitude has a significant positive correlation with forgiveness, self-forgiveness, others forgiveness, situational forgiveness, spirituality, self-discovery, relationships, eco-awareness, meaning in life, mental health, and psychological well-being; moreover, it has a significant inverse correlation with psychological distress. Forgiveness (with subscales: self, others, situational) has a significant positive correlation with spirituality, self-discovery, relationships, eco-awareness, meaning in life, mental health, and psychological well-being; moreover, it has a significant negative correlation with psychological distress. Spirituality (with subscales: self-discovery, relationships, eco-awareness) has a significant positive correlation with meaning in life, mental health, and psychological well-being; moreover, it has a significant negative correlation with psychological distress. Meaning in life has a significant positive correlation with mental health and psychological well-being, and a significant negative correlation with psychological distress. 

### 3.3. Structural Equation Modelling (Hypothesis 3)

**Figure 1:**
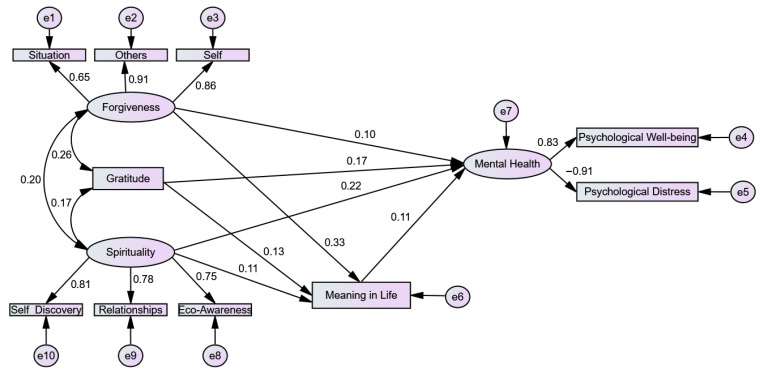
Standardized regression coefficients of structural model.

After confirming satisfactory results for the measurement model, the structural model was tested using CB-SEM to examine meaning in life as a mediator in the association of gratitude, forgiveness, and spirituality with mental health. In Table 1, the overall model fit indices are presented and overall model was χ^2^ (38, N = 1527) = 90.06, *p* < 0.01. The RMSEA and SRMR values were 0.04 and 0.03, respectively, while the CFI, GFI, and TLI were 0.99, 0.99, and 0.98. The χ^2^/df ratio was 2.34, indicating an excellent model fit. These indices collectively suggest that the model fits the data very well, as illustrated in Figure 1.

After confirming the model fit, estimates were examined to assess the direct and indirect effects of gratitude, forgiveness, and spirituality on mental health through meaning in life. The analysis was conducted using 5000 bootstrapped samples (Hayes, 2017). Table 3 presents the standardized estimates for the effects of gratitude, forgiveness, and spirituality on meaning in life and mental health.

The results indicated that gratitude was a significant positive predictor of both meaning in life (B = 0.130, *p* < 0.001) and mental health (B = 0.170, *p* < 0.001). Similarly, forgiveness was found to be a significant positive predictor of meaning in life (B = 0.330, *p* < 0.001) and mental health (B = 0.103, *p* < 0.001). Spirituality also emerged as a significant positive predictor of meaning in life (B = 0.107, *p* < 0.001) and mental health (B = 0.222, *p* < 0.001). Moreover, meaning in life significantly and positively predicted mental health (B = 0.106, *p* < 0.001).

The results of the direct and indirect effects, presented in Table 4, confirmed that meaning in life served as a significant partial mediator in the relationship between gratitude and mental health. Similarly, meaning in life was found to be a significant partial mediator in the relationship between forgiveness and mental health. Likewise, meaning in life also significantly and partially mediated the relationship between spirituality and mental health.

## 4. Discussion

This study investigated the relationship of gratitude, forgiveness, and spirituality with mental health among Pakistani postgraduate university students and examined the mediating role of meaning in life, offering new insights within this particular context. 

Regarding Hypothesis 1 (H1), results confirmed a significant positive relationship of gratitude, forgiveness, and spirituality with psychological wellbeing among postgraduate students. Numerous previous studies align with this finding, identifying gratitude, forgiveness, and spirituality as predictors of both mental health and well-being (Głaz, 2019; Javaheri et al., 2022; Zhang et al., 2018). Gratitude interventions have shown lasting positive impacts on mental health by enhancing individuals’ appreciation of life (Bohlmeijer et al., 2021) and allowing individuals to adopt a more positive outlook (Hill et al., 2013; Tolcher et al., 2024).

In the local context, Hermaen and Bhutto (2020) also found forgiveness to be positively related to well-being among Pakistani university students. Forgiveness may promote mental health and well-being by promoting coping strategies, emotional regulation, and personal growth (Aricioglu, 2016). Likewise, spirituality demonstrated a significant positive relationship with mental health and psychological well-being (D. R. Brown et al., 2013; Leung & Pong, 2021; Yoo et al., 2022). Students with higher levels of spirituality tend to report better life satisfaction and quality of life (Moreira-Almeida et al., 2014; Perez et al., 2021). Spirituality helps individuals find meaning, fosters emotional resilience, and promotes virtues such as patience, compassion, and honesty, which in turn support psychological well-being. Postgraduate education represents a transitional phase marked by heightened uncertainty regarding employment, financial stability, and familial responsibilities (Morris, 2021). 

In the specific context of Pakistani postgraduate students, these findings take on particular cultural significance. Pakistan’s society is predominantly collectivist and religiously oriented, where gratitude and spirituality are often socially reinforced values (Ali et al., 2018). Within this framework, expressions of gratitude and forgiveness are not only personal virtues but also culturally embedded mechanisms of maintaining harmony, respect, and social cohesion. For students facing intense academic and social expectations, these traits may therefore serve as adaptive resources, helping them cope with stress, maintain emotional balance, and preserve a sense of belonging. 

As regards Hypothesis 2 (H2), which proposed a negative relationship between gratitude, forgiveness, and spirituality and psychological distress, results confirm the hypothesis, consistently with extensive literature indicating that these positive traits buffer individuals against psychological difficulties. Gratitude has been found to have an inverse association with anxiety, depression, and stress (Mason, 2019; Wood et al., 2010). Forgiveness has also been shown to inversely relate to suicidal ideation, anger, and depression (Chung, 2016; Cleare et al., 2019): it serves as a powerful psychological buffer by fostering emotional healing and facilitating psychological adjustment. The findings of this study align with Li et al. (2020), who also reported an inverse relationship between forgiveness and psychological distress among university students. Spirituality, as highlighted by Chakradhar et al. (2023), is inversely associated with psychological distress: students with strong spiritual beliefs are better equipped to manage life’s challenges, which in turn reduces their vulnerability to depression, anxiety, and other psychological difficulties (Chaves et al., 2015; Pillay et al., 2016). 

Beyond confirming these findings, it is important to note that Pakistani students often face multiple psychosocial stressors, such as economic pressure, competitive academic environments, and limited access to mental health support (Yasmin et al., 2018). In this context, gratitude, forgiveness, and spirituality may function as culturally congruent coping strategies that provide emotional containment and meaning when formal psychological resources are scarce. These positive traits may also contribute to a collective sense of solidarity, encouraging empathy and mutual support among students who share similar struggles.

The results for Hypothesis 3 (H3) confirmed that meaning in life significantly and partially mediates the relationship between gratitude, forgiveness, and spirituality and mental health. This finding is consistent with earlier research, which highlights the role of meaning in life in promoting psychological resilience and reducing distress. Individuals who perceive a strong sense of meaning tend to experience improved well-being and reduced psychological difficulties (Arslan et al., 2022; Kleftaras & Psarra, 2012). Conversely, a lack of meaning has been linked to psychological dysfunction, including depression and high-risk psychopathological symptoms (Maccallum & Bryant, 2020). Recent research on Pakistani postgraduate students (Yasmin et al., 2018) has highlighted that, in Pakistan, there has been a significant increase in enrollment in postgraduate programs over the past decade. However, students face numerous challenges, including situational barriers (financial problems, lack of funding, and time management difficulties); institutional barriers (inefficient university procedures, inadequate support services, poor infrastructure); dispositional barriers (low self-esteem, health issues, poor communication skills); and academic barriers (limited writing, information processing, and IT skills, as well as insufficient teacher competencies). The capacity to find meaning in such challenging situations may be crucial to sustain both academic performance and psychological well-being of postgraduate students. 

To note, in collectivist and strongly religious contexts such as Pakistan, spirituality and meaning in life may partially overlap in individuals’ lived experiences, as spiritual beliefs often provide a central framework for articulating life purpose and meaning. However, acknowledging this cultural overlap does not undermine their analytical distinction; instead, it highlights how spirituality and meaning in life may mutually reinforce one another in shaping mental health outcomes within specific socio-cultural contexts. This distinction is supported by the measurement model, which demonstrated adequate discriminant validity, and by the structural analyses showing that spirituality exerted both direct effects on mental health and indirect effects through meaning in life. Future cross-cultural studies could further explore these relationships in contexts with a more individualistic orientation and/or a more secular approach, to examine whether the role of gratitude, forgiveness, and spirituality as coping resources operates similarly or differently across diverse cultural settings.

In a context where higher education is both a personal aspiration and a collective expectation, students often experience a dual tension between self-development and family duty (Ali et al., 2018). Gratitude and spirituality may enable them to reconcile these tensions, transforming academic stress into a sense of purpose and contribution. Forgiveness, meanwhile, may buffer the relational strains that arise in competitive or hierarchical university environments. Together, these self-transcendent traits allow students to reinterpret challenges as meaningful steps within their broader life narrative—a process central to psychological resilience in collectivist societies.

This research offers several noteworthy strengths. It addresses a significant gap in the Pakistani context, particularly among postgraduate students in public sector universities in Punjab, where limited prior research has examined the relationships among gratitude, forgiveness, spirituality, and mental health. By providing empirical evidence on these associations and the mediating role of meaning in life, the study makes a valuable theoretical contribution to the literature on positive psychology. Practically, the findings can guide university administrators and policymakers in designing mental health initiatives that incorporate gratitude, forgiveness, spirituality, and meaning-making as integral components of student support services. Beyond the local context, these results have global relevance, offering insights for researchers and practitioners in other regions seeking to understand and promote student mental health through the lens of positive psychology.

The findings of this research have significant practical implications for clinical psychologists, mental health professionals, university administrators, and policymakers. The results highlight the importance of integrating positive psychological constructs—gratitude, forgiveness, spirituality, and meaning in life—into mental health initiatives on university campuses. Universities should consider offering dedicated programmes, workshops, or courses in positive psychology to foster these traits, which have been shown to promote well-being and reduce psychological distress among students.

Given the centrality of spirituality and community in Pakistani culture, interventions could integrate faith-sensitive counseling and group-based meaning-making activities that align with students’ values. Additionally, postgraduate mentorship programs could foster gratitude and forgiveness through reflective dialogues, helping students translate academic challenges into personal growth experiences. The Higher Education Commission (HEC) of Pakistan should also take a proactive role by encouraging or mandating the inclusion of positive psychology content across curricula at the undergraduate and postgraduate levels. Embedding these principles into various academic disciplines can equip students with effective coping strategies and improve their overall psychological resilience.

Currently, most Pakistani universities lack formal coursework or syllabus aimed at addressing students’ psychological well-being. Psychology departments and student support units should be encouraged to offer free psychological counseling services on campus, enabling students to access professional help without stigma or financial burden. These services could include training sessions on emotional regulation, stress management, and the development of gratitude, forgiveness, and spiritual practices.

From an applied perspective, the present findings suggest the central mediating role of meaning in life as a key leverage point for university-based mental health interventions. Rather than targeting gratitude, forgiveness, and spirituality in isolation, interventions may be most effective when they explicitly aim to strengthen students’ sense of meaning, coherence, and purpose, using these positive orientations as pathways through which meaning can be cultivated. For example, university workshops, counseling services, or reflective group activities could be designed to support meaning-making processes by encouraging gratitude-based reflection, forgiveness-related reappraisal of interpersonal experiences, and spiritually sensitive exploration of values and purpose. Such meaning-centered initiatives may help postgraduate students interpret academic challenges within a broader life framework, thereby enhancing psychological well-being and reducing distress.

Although the present findings were consistent across the overall sample, it is important to consider that unmeasured demographic factors—such as gender, socioeconomic status, and rural–urban background—may shape how gratitude, forgiveness, spirituality, and meaning in life are experienced and mobilized among postgraduate students, especially in the Pakistani context, where gender norms, economic constraints, and disparities between rural and urban educational environments may influence access to psychological resources, modes of meaning-making, and exposure to stressors, potentially conditioning the strength or direction of the observed relationships. Despite its contributions, this study has a few limitations. Data were collected only from one province (Punjab), which limits the generalizability of the findings to all Pakistani university students. The research focused exclusively on full-time postgraduate students in public sector universities and did not address mental health issues in private universities or undergraduate students. Although demographic variables such as gender, socioeconomic status, and rural–urban background were collected, they were not included as moderators in the present analyses. This decision was made to maintain parsimony and analytical clarity, as the primary aim of the study was to test a theoretically driven mediation model centered on meaning in life. Future research could extend the present model by conducting exploratory multigroup structural equation modeling analyses, that would be particularly valuable in contexts like Pakistan, where social and structural inequalities may influence students’ psychological functioning. While the present study focused on the hypothesized relationships, future research could examine alternative models, including possible reverse or reciprocal associations between the variables. To note, although SEM was employed to test the proposed mediation model, the cross-sectional design of the study limits causal inference. Mediation analysis was conducted with cross sectional data reflect statistical associations among variables rather than temporal or causal process. Therefore the directionality of relationships cannot be conclusively established. Future research using longitudinal or experimental designs are recommended to more rigorously examine the causal mechanisms underlying these associations. Moreover, future studies could adopt longitudinal or mixed-method designs to examine how meaning in life evolves across different postgraduate stages or to capture the lived experiences of how gratitude and spirituality are expressed and practiced in Pakistani cultural settings. Such approaches would deepen understanding of the ways in which positive psychology traits interact with cultural identity, faith, and educational stressors.

## 5. Conclusions

This research concludes that mental health is significantly associated with gratitude, forgiveness, and spirituality among postgraduate university students in Pakistan. Students who demonstrated higher levels of these positive traits reported lower psychological distress and greater psychological well-being. Furthermore, meaning in life was found to partially mediate the relationship between gratitude, forgiveness, and spirituality and mental health, highlighting its critical role in enhancing students’ psychological functioning. Overall, these findings emphasize the value of promoting positive psychological traits and meaning-making among students to improve their mental health. The results can inform interventions, curriculum development, and policy decisions aimed at supporting university students’ emotional and psychological well-being. Specifically, universities may translate these insights into practical initiatives such as workshops, group-based activities, or counseling services designed to foster gratitude, forgiveness, spirituality, and meaning-centered reflection among postgraduate students.

## Figures and Tables

**Table 1:** Model Fit Indices of Measurement and Structural Model.

Model	χ^2^/df	GFI	CFI	NFI	RMSEA	SRMR
Measurement Model	2.93	0.91	0.90	0.92	0.03	0.03
Structural Model	3.34	0.99	0.98	0.98	0.04	0.02

Note: χ^2^ > 0.05. Goodness of fit index (GFI), comparative fit index (CFI), normed fit index (NFI), root mean square error of approximation (RMSEA), Standardized root mean square (SRMR).

**Table 2:** Pearson Product Moment Correlation Analysis of Study Variables [N = 1527].

Variables	1	2	2a	2b	2c	3	4	4a	4b	4c	5	5a	5b
1. Gratitude	-	0.237 **	0.232 **	0.231 **	0.156 **	0.159 **	0.135 **	0.125 **	0.146 **	0.234 **	0.246 **	0.236 **	−0.227 **
2. Forgiveness		-	-	-	-	0.190 **	0.117 **	0.133 **	0.198 **	0.368 **	0.203 **	0.166 **	−0.208 **
2a. Self			-	0.683 **	0.549 **	0.182 **	0.130 **	0.156 **	0.170 **	0.349 **	0.177 **	0.153 **	−0.176 **
2b. Others				-	0.596 **	0.173 **	0.104 **	0.126 **	0.178 **	0.332 **	0.197 **	0.167 **	−0.198 **
2c. Situation					-	0.142 **	0.072 **	0.064 *	0.170 **	0.281 **	0.157 **	0.114 **	−0.171 **
3. Spirituality						-	-	-	-	0.201 **	0.250 **	0.212 **	−0.251 **
3a. Self-Discovery								0.634 **	0.600 **	0.110 **	0.216 **	0.171 **	−0.226 **
3b. Relationships								-	0.575 **	0.155 **	0.191 **	0.159 **	−0.194 **
3c. Eco-Awareness									-	0.207 **	0.230 **	0.202 **	−0.227 **
4. Meaning in Life										-	0.215 **	0.198 **	−0.205 **
5. Mental Health											-	-	-
5a. Psychological Well-being													−0.760 **
5b. Psychological Distress													-
Mean	27.77	82.70	27.64	27.37	27.69	86.6948	14.84	22.63	49.23	44.33	116.24	48.50	86.25
SD	8.71	25.41	10.01	9.71	9.36	23.68	5.17	7.10	14.93	15.57	45.99	20.50	28.43
Median	30.00	90.00	30.00	30.00	31.00	89.0000	16.00	24.00	52.00	48.00	119.00	49.00	85.00

** *p* < 0.01; * *p* < 0.05.

**Table 3:** Standardized Estimates of Gratitude, Forgiveness, and Spirituality on Meaning in Life and Mental Health.

Variables	Meaning in Life			Mental Health		
	Β	SE	*p*	Β	SE	*p*
Gratitude	0.130 **	0.044	0.000	0.170 **	0.054	0.000
Forgiveness	0.330 **	0.072	0.000	0.103 **	0.086	0.000
Spirituality	0.107 **	0.037	0.000	0.222 **	0.047	0.000
Meaning in Life	-	-	-	0.106 **	0.031	0.000

** *p* < 0.001.

**Table 4:** Standardized Estimates of Direct and Indirect Effects of the Paths.

Variables	Meaning in Life		Mental Health	
	Direct	Indirect	Direct	Indirect
Gratitude	0.130 **	-	0.170 **	0.014 **
Forgiveness	0.330 **	-	0.103 **	0.035 **
Spirituality	0.107 **	-	0.222 **	0.011 **
Meaning in Life	-	-	0.106 **	-

** *p* < 0.001.

## Data Availability

Data will be made available upon reasonable request to the corresponding author.

## Supplementary Materials

The following supporting information can be downloaded at: https://www.mdpi.com/article/10.3390/ejihpe16020025/s1, Table S1: Demographic Information Profile.
