# Supplementary material for: Meaning in Life Mediates Associations Between Gratitude, Forgiveness, Spirituality, and Mental Health in Postgraduate Students
Source: Eur J Investig Health Psychol Educ. 2026 Feb 13;16(2):25. doi: 10.3390/ejihpe16020025 (PMC12939757; doi:10.3390/ejihpe16020025)
Supplement: Supplementary file 1 [file ejihpe-16-00025-s001.zip › ejihpe-3951900-supplementary.pdf]

Supplementary Materials

Table S1. Demographic Information Profile

Frequency Distribution of Demographic Sheet (N=1527)

Respondent's Characteristics		*f (%)*	*M(SD)*
Age			24.89(2.21)
Gender	Male	795 (52.20)	
	Female	732 (47.80)	
Education	Master	1383 (90.60)	
	PhD	144 (9.40)	
University	University of Punjab	151 (9.90)	
	Lahore College for Women University	137 (9.00)	
	Government College University Faisalabad	155 (10.20)	
	Arid Agriculture University	171 (11.20)	
	University of Gujrat	166 (10.90)	
	University of Sahiwal	171 (11.20)	
	Islamia University of Bahawalpur	161 (10.50)	
	Bahauddin Zakariya University	138 (9.00)	
	University of Sargodha	150 (9.80)	
	Ghazi University	127 (8.30)	
Faculty	Faculty of Science	507 (33.20)	
	Faculty of Arts and Humanities	513 (33.60)	
	Faculty of Social Sciences	507 (33.20)	
Departments	Mathematics	170 (11.10)	
	Physics	178 (11.70)	
	Chemistry	158 (10.30)	
	English	161 (10.50)	
	Urdu	180 (11.80)	
	Islamic Studies	173 (11.30)	
	Psychology	170 (11.10)	
	Economics	171 (11.20)	
	Sociology	166 (10.90)	
Family Monthly Income (in Pakistani Rupees)			70355.60 (40838.43)
Home Residence	Urban	615 (40.30)	
	Rural	912 (59.70)	
Marital Status	Unmarried/Single	1251 (81.90)	
	Married	268 (17.60)	
	Divorced	08 (0.50)	
Family System	Separate	782 (51.20)	
	Joint	745 (48.80)	
